# Modelling and impact analysis of interdependent characteristics on cascading overload failure of syncretic railway networks

**DOI:** 10.1371/journal.pone.0239096

**Published:** 2020-09-21

**Authors:** Su Liu, Chengshuang YIN, Dingjun Chen, Shaoquan Ni

**Affiliations:** 1 School of Transportation and Logistics, Southwest Jiaotong University, Chengdu, China; 2 National and Local Joint Engineering Laboratory of Comprehensive Intelligent Transportation, Southwest JiaoTong University, Chengdu, China; 3 National Engineering Laboratory of Integrated Transportation Big Data Application Technology, Southwest JiaoTong University, Chengdu, China; Tongii University, CHINA

## Abstract

To study the performance and mutual influence of a syncretic railway network (SRN) that comprises high-speed railway, regional railway, and urban rail transit under the condition of traffic overload during peak hours, we discuss the interdependent characteristics on cascading overload failure of SRNs under the cooperative organization from the perspective of an interdependent network. However, most existing research on cascading failure in interdependent network ignores the inconsistency between the physical structure and transportation organization of the subnetwork in an actual network, in addition to the restrictions on the load redistribution strategy of stations and sections in the load-capacity model of the interdependent network; especially, the influence of transfer behavior on the load redistribution inter subnetwork. In this study, we investigate the robustness of an interdependent SRN under overload and risk propagation. We propose a partially interdependent network model of a multimode rail transit, develop a novel cascading overload failure model with tunable parameters of load redistribution inter subnetwork, and analyze interdependent characteristics, cascade failure process, and robustness of an SRN under multiscene conditions, i.e., different attack and load distribution strategies, via simulations. A case study of an SRN in the metropolitan area of Chengdu, China is presented; the results indicate that, when the reserve coefficient of the metro subnetwork is 0.4 and the overload coefficient of the regional railway subnetwork is greater than 1.2, the station reserve capacity and overload capacity of the SRN is appropriately improved. When passenger load increases to a certain range, the reserve and overload capacities of stations in the regional railway subnetwork do not considerably contribute to robustness. Thus, a surplus load distribution strategy is recommended to improve robustness. The results of this paper have considerable significance for the planning, structural optimization, and operation safety of SRNs.

## Introduction

With an increase in urban agglomeration in China and the progress in rail transit technology related to construction, multimode rail transit systems have played an important role in the economic spatial connection of urban agglomerations. The physical connection or logical function among multimode rail transit shares an interdependent relationship because of collinear or transfer modes. Hence, syncretic railway network (SRN) functions are complex, and there is a considerable diversity in passenger flow demand. The phenomenon of morning and evening peaks is prominent in urban rail transit systems. During peak hours, some high-speed and regional railways have organized trains to stop station-by-station within the metropolitan area to complement urban rail transit and support urban transportation partially. When an SRN is in an abnormal circumstance such as a sudden increase in passenger flow or deliberate terrorist attacks, some stations are disturbed by internal and external factors that result in overload or failure; this leads to changes in passenger flow volume (PFV) in these affected stations. Thus, the PFV of other rail transit network will be affected correspondingly through the transfer station. This can lead to the collapse of most or the entire SRN, which would seriously affect service quality. This phenomenon is called the cascading failure of the SRN. In particular, the cascading overload failure is more likely to occur during peak hours. Our aim is to determine the interdependent characteristics involved in the cascading failure of the SRN to show that the removal or overload of one node can have serious consequences on the entire network. This is an important theoretical basis for the safety and control management of SRNs to design propagation mechanisms and analyze critical stations under multiscenario conditions.

Many researchers have already investigated cascading failure and robustness of networks. In general, cascading failures are categorized into two types: failure of the network under physical or logical connection, and the cascading failure of complex network with load variation. For the first type, most relevant literatures do not consider the load of nodes or edges. The state of a node is closely related to that of its neighbors; when a node fails, the state transition of the node can lead to a state transition of its neighboring nodes with a certain probability. Studies on this type of cascading failure is mostly based on the cascading failure of a theoretical networks such as scale-free networks, small world networks, and ER networks, and they adopt a percolation model, fiber-bundle model, etc., to compare the robustness of different networks [1–3]. In general, the capacity limitation of nodes and edges should be considered in load distribution. When the structure of a network changes, the load redistribution strategy makes it complicated to ensure the robustness of complex networks. Most research on the second type of cascading failure adopts an analytic method and a simulation method. Previous studies [4–7] proposed a load-capacity model to describe the cascades of failures. Because most actual networks are not isolated, there are interactions between different networks; therefore, research on the cascading failure of interdependent network considers the coupling relationship between networks based on the original problems of single-layer networks. The results of the studies show that, only a small number of nodes in an interdependent network can lead to collapse unlike that in a single-layer complex network. Further, research on the cascade failure of interdependent network is based on percolation and load-capacity models. Earlier studies focused on a cascading model with different attack strategies and coupling modes of interdependent networks [8–14]. Further, some previous studies focused on a cascading failure model of interdependent networks with groups, multiple dependency relations, and cliques [15–17]. Following these works, several studies employed the cascading failure model on weighted urban traffic networks [18, 19].

To consider the complexity of travel behaviors, the effect of cascading failures was determined from different perspectives. Liu et al. [20, 21] simulated the effects of different travel prior experiences and travel information using a Bayesian network and hybrid utility inference to the road network properties and cascading failures. Previous studies [22–26] analyzed the effect of cascading failure on urban road traffic and rail transit networks to evaluate the scope of the influence, identify the source of the influence, and analyze the capacity of urban road network and the mitigation strategies for cascading failures. Furthermore, cascade failure in multilevel transportation networks, i.e., bus-metro syncretic network and passenger traffic networks of urban agglomeration have also been studied [27, 28].

Although previous researchers focused on a single urban road traffic network or metro network, in reality, different modes of transportation are more cooperative. When the problem dimension is reduced by building a weighted network, interdependent characteristics between different traffic networks are ignored. However, according to its physical and operation characteristics, the SRN is an asymmetric multiple interdependent network with an inconsistent number of sub-network nodes and fewer interdependent nodes; thus, the structure and performance of the SRN is complex. To study the interdependent influence of the SRN under the condition of overload during peak hours, we discuss the cascading overload failure of the SRN under the cooperative organization from the perspective of interdependent network. However, most existing research ignores the restrictions on the load redistribution strategy of stations and sections in the load-capacity model of an interdependent network, especially the influence of transfer behavior on the load redistribution inter subnetwork; further, subnetworks adopt different load redistributions in the actual network. To this end, we introduce a cascading overload failure model with tunable parameters of the reserve coefficient, overload coefficient, and transfer coefficient to explore the interdependent characteristics and robustness of the SRN, and we explore the failure mechanism with different load redistributions of subnetworks; we consider the inconsistency of the physical structure and transportation organization of the subnetwork. The method proposed in this paper can analyze the interdependent characteristics and robustness of the SRN, and it is therefore an essential tool for proactive risk and crisis management in the integrated operation of rail transit.

The rest of this paper is organized as follows: The interdependent SRN model and cascading overload failure model of the SRN are presented in Section 2. Section 3 provides the analysis of the interdependent characteristics and robustness of the SRN in the metropolitan area of Chengdu. Finally, the conclusions are summarized in Section 4.

## Cascading overload failure model of interdependent SRN

### Interdependent SRN model

The cascading failures of the SRN can be divided into implicit (caused by load redistribution) and explicit (caused by structural failures) failures. The cascade overload failure of the SRN is an implicit failure. Accordingly, the following assumptions and definitions are considered to propose an interdependent SRN model.

- The infrastructure network of the SRN is an unweighted undirected network.
- The service network of the SRN is a directed network and it includes both the up and down directions.
- The transfer of passenger flow between subnetworks has directionality. A bidirectional association exists between subnetworks.
- Only the initial node is invalidated by the attack. The other stations operate under three statuses: normal, full load and overload failed. All sections are in normal operation.
- There is at least one interdependent node between different subnetworks.

Networks A and B can be set according to an actual scenario. We consider the metro subnetwork (MN) as network A and a regional railway subnetwork (RRN) as network B expressed as *G*_*A*_ = (V_*A*_,E_*A*_) and *G*_*B*_ = (V_*B*_,E_*B*_), respectively; where V_*A*_ = {1,2,⋯*N*_*A*_}, E_*A*_ = {*e*_*ij*_}, V_*B*_ = {1,2,⋯*N*_*B*_}, and E_*b*_ = {*e*_*ij*_} are the set of stations and sections in the MN and RRN, respectively. Further, *N*_*A*_ and *N*_*B*_ denote the number of nodes in the MN and RRN, respectively. If there is connection between stations in the single network, *e*_*ij*_ = 1, otherwise *e*_*ij*_ = 0. We define the interdependent correlation matrix of an SRN as *R*. If the logical relationship of an interdependent node $V_{m_{A}}$ or $V_{m_{B}}$ has a bidirectional association, $R_{m_{A}m_{B}}=1$, otherwise $R_{m_{A}m_{B}}=0$. The set of interdependent correlations can be expressed as

$$R=\left\{R_{m_{A}m_{B}}=(V_{m_{A}},V_{m_{B}})|m_{A}=1,2,\cdots N_{A};m_{B}=1,2,\cdots N_{B}\right\}$$

Accordingly, the SRN can be denoted as *G* = *G*_*A*_∪*G*_*B*_∪*R*.

### Model of cascading overload failure

**Fig 1:**
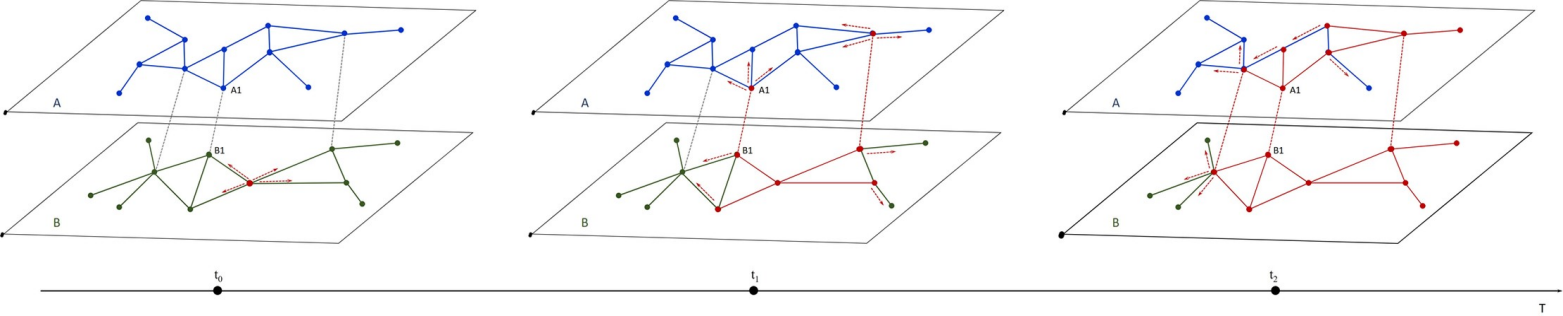
Cascade overload failure of the SRN.

According to the connection and transfer characteristic, stations can be expressed as interdependent and conventional nodes. The dynamic process of cascading overload failures in SRNs is shown in Fig 1. At the initial state, the PFV of stations and sections in the SRN is less than the capacity, and the SRN is considered under normal operation. When a station in the SRN overloads or fails, the passengers originally in the station need to re-plan their travel paths. This leads to two scenarios: overload and failure. The overload phenomenon implies that the passenger flow exceeds the capacity of the station within a certain period. At this time, the station limits the passengers entering the station, and the passengers in the station may not be able to board the train. Therefore, at this time, the train passes through the station and only a small part of the passenger flow can be transferred. The phenomenon of failure implies that the infrastructure of the station is damaged and the transfer of passenger flow inside and outside the station cannot be achieved, and therefore, the operation efficiency is nearly zero when a station fails. In this case, the passengers will choose the station that is close to the affected station with sufficient capacity or transfer to other modes of rail transit. Thus, when a station in network A is attacked or overloaded, it is necessary to determine whether the station is an interdependent node. If it is an interdependent node, the load will be distributed according to the load redistribution strategies among the intra and inter networks, respectively. If the station is only a conventional node in a single subnetwork, the load of the station will be transferred to its adjacent normal nodes according to a different internal load redistribution strategy. After load redistribution, if the total of the additional load allocated to the adjacent nodes and its initial load does not exceed the capacity, the network operates normally. If not, the adjacent nodes may overload or fail, which can result in a further redistribution of load. This is attributed to the fact that, when the passenger flow is greater than the capacity of stations, the stations limit flow within a certain period of time. Although the station overloads and needs to limit passenger flow, the trains continue to run as planned, and the operation efficiency remains valid to some degree.

#### Initial state

*Initial load and capacity*. The initial load of stations in the SRN is the PFV of the station during the peak hours. All stations initially operate normally and have initial loads *L*_*i*_ determined as $L_{i}=\Phi_{1}w_{i}+\Phi_{2}w_{i}^{'}$. Here, *w*_*i*_ and $w_{i}^{'}$ is the PFV of the metro station and the railway stations, respectively. Φ_1_ and Φ_2_ are Boolean variables. If the station is a metro station, Φ_1_ = 1, otherwise Φ_1_ = 0. If the station is a railway station, Φ_2_ = 1; otherwise Φ_2_ = 0. If the station is a transfer station between the MN and the RRN, Φ_1_ = Φ_2_ = 1. The initial load of the section between adjacent stations is defined as *L*_*ij*_ = *w*_*ij*_, and *w*_*ij*_ is the PFV of sections. In general, interdependent SRN stations have reserve capacity as per design, which can be improved further by the expansion and reconstruction of the stations in operation. Therefore, the capacity of the SRN is related to the predicted PFV and reserve capacity. The capacity *c*_*i*_ of station *i* is proportional to its initial load *L*_*i*_, and it is defined as *c*_*i*_ = (1+*β*)*w*_*i*_, where *β* (*β*≥0) is a tunable parameter of the reserve coefficient. In addition, the larger the value of *β*, the larger is the reserve capacity of the station, and the more robust is the SRN. For the sake of simplicity, the capacity of adjacent sections in the MN and RRN is set as a certain value in this study and it is denoted as *c*_*ij*_ and $c_{ij}^{'}$, respectively.

*Status of stations*. There are two reasons for the failure of an SRN. One is physical failure caused by failed infrastructure, and the other is a functional failure caused by an overload failure. However, when the PFV is greater than the capacity of the station, the station does not necessarily fail. The capability of stations can be improved in a short time by employing a passenger transport organization method, i.e., the overload capacity of the station. When a station fails because of overload, the physical structure (i.e., station and sections between adjacent stations) are normal; however, the overloaded station will no longer accept additional load. Thus, the logical connection status of this station is disconnected, which results in a logically isolated node. Thus, the status of stations is related to PFV, additional load, reserve capacity, overload capacity, physical connection status, and logical connection status. The status *S*_*i*_(*t*) of station *i* at time *t* can thus be expressed as

$$S_{i}\left(t\right)=\{\begin{array}{ll} 1 & L_{i}(t)\leq c_{i}\cap k(i)\neq 0\\ overload & c_{i}<L_{i}(t)<\alpha c_{i}\cap k(i)\neq 0\\ 0 & L_{i}(t)\geq \alpha c_{i}\cup k(i)=0 \end{array}$$ (1)

where *L*_*i*_(*t*) is the passenger load of station *i* at time *t*, and *α* (*α*>1) is a tunable parameter that represents the overload coefficient of stations. The maximum overload of the station is *αc*_*i*_; and *k*_*i*_ is the logical connectivity of station *i*. If adjacent stations overload, station *i* will not distribute additional load to them, and the logical connection is disconnected. If *L*_*i*_(*t*)≤*c*_*i*_ and without a disconnected station, station *i* is in normal operation, *S*_*i*_(*t*) = 1. If *c*_*i*_<*L*_*i*_(*t*)<*αc*_*i*_ and without a disconnected station, station *i* is in overload. Otherwise, *S*_*i*_(*t*) = 0.

#### Attack strategy

This paper discusses the effect of overload and failure of stations on interdependent SRN, and the difference between conventional stations and interdependent stations with overload failure. According to the characteristics of SRNs, eight attack strategies are adopted. Among them, hierarchical attack is a strategy wherein interdependent nodes are selected to attack first, followed by attacking conventional intra nodes. The eight attack strategies are listed in Table 1.

**Table 1:** Eight attack strategies.

No.	Attack strategy	No.	Attack strategy
**1**	High betweenness attack strategy of the MN	**5**	Simultaneous attack strategy based on load
**2**	Hierarchical attack strategy based on betweenness of the MN	**6**	High betweenness attack strategy of the RRN
**3**	High load attack strategy of the MN	**7**	Hierarchical attack strategy based on betweenness of the RRN
**4**	Simultaneous attack strategy based on betweenness	**8**	High load attack strategy of the RRN

#### Load redistribution strategy

In the existing cascade failure model, local load redistribution strategies of the overload node include average distribution, degree distribution, surplus load distribution, etc. For a cascading failure model of traffic network [22], global load redistribution strategies such as user equilibrium assignment and system optimization assignment are proposed. However, the cascading overload failure of stations in the SRN is caused by station characteristics. It is unreasonable to employ the load redistribution strategy as a global load redistribution strategy because of factors such as distance, travel time, and cost. Further, different modes of rail transit adopt different load redistribution strategies.

#### Load redistribution strategy intra subnetwork

*Strategy 1*: *Surplus Load Distribution (SLD)*. When the station of a single subnetwork distributes the load to an adjacent station considering the surplus capacity of the station and the section connected to adjacent stations that are disturbed (attacked or overload), the SLD strategy [28] is adopted to redistribute the load intra subnetwork. Because of capacity limitations, the larger the surplus capacity, the more additional load it can accept. If station *i* distributes load to the adjacent stations after being attacked or after overloading, the load redistribution probability of adjacent station *j* at time *t* is *P*_*ij*_(*t*), which can be expressed as

$$P_{ij}\left(t\right)=\{\begin{array}{ll} \frac{c_{ij}-L_{ij}\left(t\right)}{\sum_{k\in A}\min\left[\left(c_{ik}-L_{ik}\left(t\right)\right),\left(c_{k}-L_{k}\left(t\right)\right)\right]} & c_{ij}-L_{ij}\left(t\right)<c_{j}-L_{j}\left(t\right)\\ \frac{c_{j}-L_{j}\left(t\right)}{\sum_{k\in A}\min\left[\left(c_{ik}-L_{ik}\left(t\right)\right),\left(c_{k}-L_{k}\left(t\right)\right)\right]} & c_{ij}-L_{ij}\left(t\right)>c_{j}-L_{j}\left(t\right) \end{array}$$ (2)

*Strategy 2*: *Distance Distribution (DD)*. Considering the cost of sections, the DD strategy is adopted to reallocate the load intra subnetwork, and the load redistribution probability *P*_*ij*_ of adjacent station *j* at time *t* is given as

$$P_{ij}\left(t\right)=\frac{1}{d_{ij}}\cdot \frac{1}{\sum_{k\in A}d_{ik}}$$ (3)

where *d*_*ij*_ is the distance between station *i* and the adjacent station *j*; *A* is the set of stations adjacent to station *i* intra subnetwork; and *j* is the station in set *A*.

*Strategy 3*: *Betweenness Distribution (BD)*. To reflect the importance of stations with different physical or logical connections in the SRN, the BD strategy is proposed to redistribute the load intra subnetwork. *P*_*ij*_ can be expressed as

$$P_{ij}\left(t\right)=b_{j}/\sum_{k\in A}b_{k}$$ (4)

where *b*_*j*_ is the betweenness of station *j*, *b*_*k*_ is the betweenness of stations adjacent to station *i*, *A* is the set of stations adjacent to station *i*, and *j* is the station in set *A*.

Let Δ*L*_*i*_(*t*+1) be the additional load redistributed by the station to the adjacent station at time *t*+1 after the SRN is disturbed (attacked or overload). Δ*L*_*i*_(*t*+1) is expressed as

$$\Delta L_{i}\left(t+1\right)=\{\begin{array}{ll} 0 & S_{i}(t)=1\\ P_{ij}\left(t\right)(L_{i}\left(t\right)-c_{i}) & S_{i}(t)=overload\\ P_{ij}\left(t\right)L_{i}\left(t\right) & S_{i}(t)=0 \end{array}$$ (5)

where if *S*_*i*_(*t*) = 1, the next moment will not distribute load to the adjacent station. If *S*_*i*_(*t*) = *overload*, *L*_*i*_(*t*)−*c*_*i*_ is distributed to the adjacent stations and no additional load is accepted at time *t*+1. Otherwise, *L*_*i*_(*t*) is allocated to adjacent stations and no additional load is accepted thereafter.

#### Load redistribution strategy inter subnetwork

If there is an interdependent relationship between node *m* of network *A* and node *n* of network *B*, the failure of node *m* will lead to a complete failure of node *n* as per the existing studies on interdependent networks. However, the failure of one rail transit station will not directly lead to a failure of the interdependent ones in the SRN. For the sake of simplicity, this paper assigns priority to the passenger flow transfer between the subnetworks of the SRN. Under this assumption, if node *m* of network *A* fails, the load of the failure node is distributed to node *n* of network *B* with an interdependent relationship based on certain rules, which result in the load change of node *n*; this can be expressed as Δ*L*_*n*_(*t*+1) = *μL*_*m*_(*t*), where *μ* denotes the tunable parameters of the passenger flow transfer inter subnetworks *μ*∈[0,1].

### Measurement indictor

Based on the network representation of the SRN, there are several measurement indictors based on network science and graph theory, such as edge connectivity, node connectivity, and size of giant connected component (GCC) [29]. The GCC is the largest connected subgraph that has the maximum number of nodes, and it constitutes a giant mutually connected component. Using percolation theory, network robustness can be investigated via the occupied fraction of the largest connected component considered as potentially functional [30, 31]. The size of the GCC has been widely adopted to measure the connectivity of a variety of real-world systems [32, 33]. When the SRN is attacked or fails locally, the size of the GCC is considered as the absolute index of robustness, and it is expressed as

$$V=\left(N_{A}^{''}+N_{B}^{''}\right)/\left(N_{A}+N_{B}\right)$$ (6)

However, this metric describes the overall significance of GCC, while ignoring the detailed connections among nodes (both those part-of and not-part-of GCC). In the SRN, parts of edges (even not in GCC) can still enable travel demand between two corresponding stations that are under operation conditions [34]. Therefore, we introduce survival rate *S* to evaluate the proportion of stations that have not failed in the SRN when cascading failure is terminated; this is described as

$$S=\left(1‐\left(F_{A}+F_{B}\right)\right)/\left(N_{A}+N_{B}\right)$$ (7)

Simultaneously, failure rate *F*, which is the proportion of failed stations in the total number of affected stations in the SRN, is defined as a metric to evaluate the robustness of the SRN. In addition, the relative indictor *F* of robustness can be formulated as

$$F=\left(F_{A}+F_{B}\right)/\left(N_{A}^{'}+N_{B}^{'}\right)$$ (8)

where $N_{A}^{''}$, $N_{B}^{''}$, *F*_*A*_, *F*_*B*_, $N_{A}^{'}$, and $N_{B}^{'}$ are the size of the GCC, the number of failed stations (attack and overload), and the number of affected stations (full load, attack, and overload) of networks *A* and *B*, respectively.

## Case analysis

**Fig 2:**
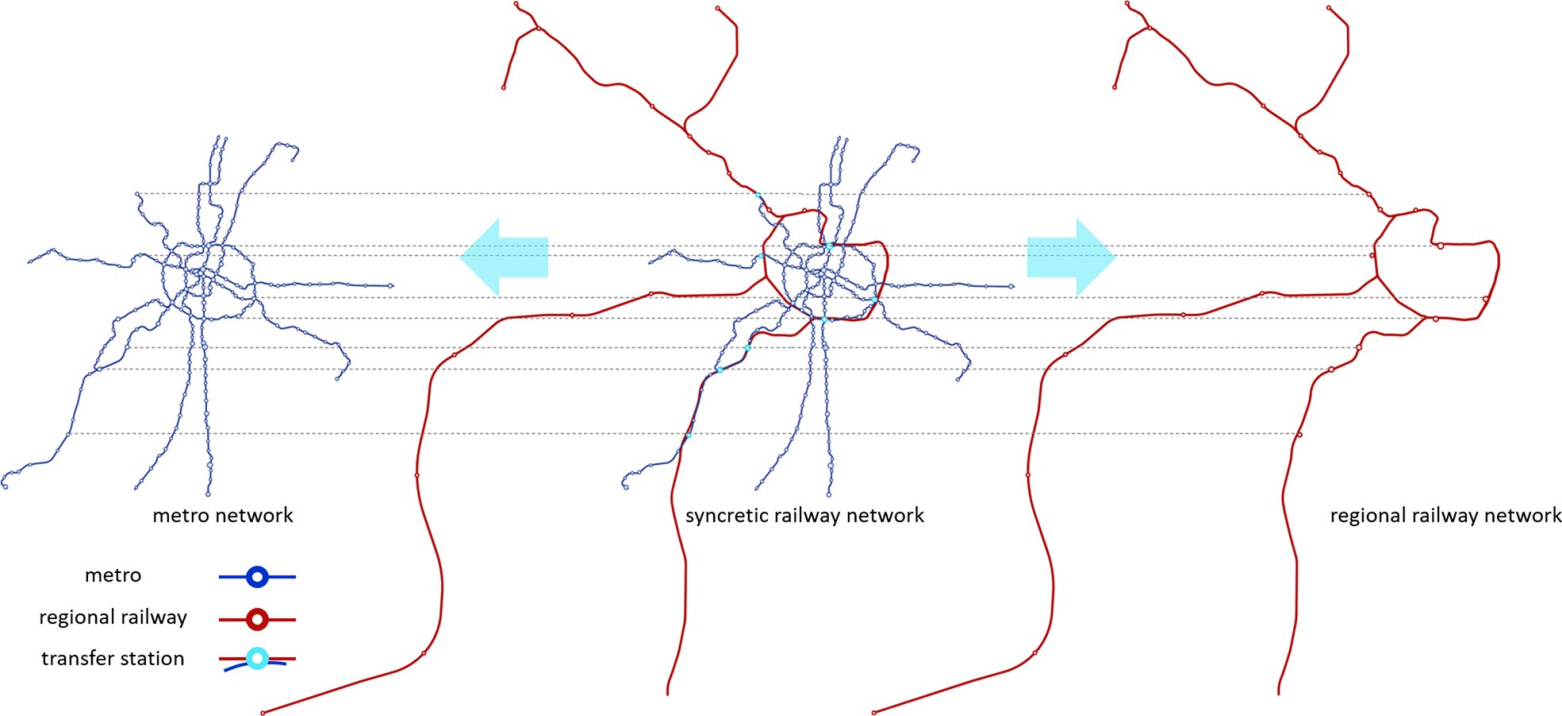
SRN in the metropolitan area of Chengdu.

An interdependent SRN in the metropolitan area of Chengdu, China is considered as an example (Fig 2). By November 2019, the MN of Chengdu includes lines 1, 2, 3, 4, 5, 7 and 10, with 202 stations in total. The RRN in the metropolitan area of Chengdu includes the line of Chengdu–Ya'an, Chengdu–Dujiangyan, and Chengdu–Leshan, with a total of 30 stations. There are 8 transfer stations between the MN and RRN, i.e., Chengdu railway station, East railway station, South railway station, West railway station, Xipu railway station, West Shuangliu railway station, Shuangliu airport railway station, and Xinjin station.

In this paper, the daily PFV of the stations in December 2019 is considered as the initial load. The top five stations of the MN and RRN with higher load and higher betweenness are listed in Table 2.

**Table 2:** Top five stations of the MN And RRN in the metropolitan area of Chengdu.

Rank	MN		RRN	
	Betweenness	Load	Betweenness	Load
**1**	South Railway Station	Chunxi Road	Chengdu Railway Station	East Railway Station
**2**	Taipingyuan	East Chengdu Railway Station	South Railway Station	South Railway Station
**3**	2^nd^ Beizhan Road West	Xipu	Anjing Railway station	Xipu Railway station
**4**	Chengdu University of TCM & Sichuan Provincial People’s Hospital	3^rd^ Tianfu Street	Xipu Railway station	Dujiangyan Railway station
**5**	Shenxianshu	Tianfu Square	Shuangliu Airport Railway Station	Leshan Railway station

### Effect of tunable parameters on the robustness of interdependent SRN

#### Effect of *α*&*β*

**Fig 3:**
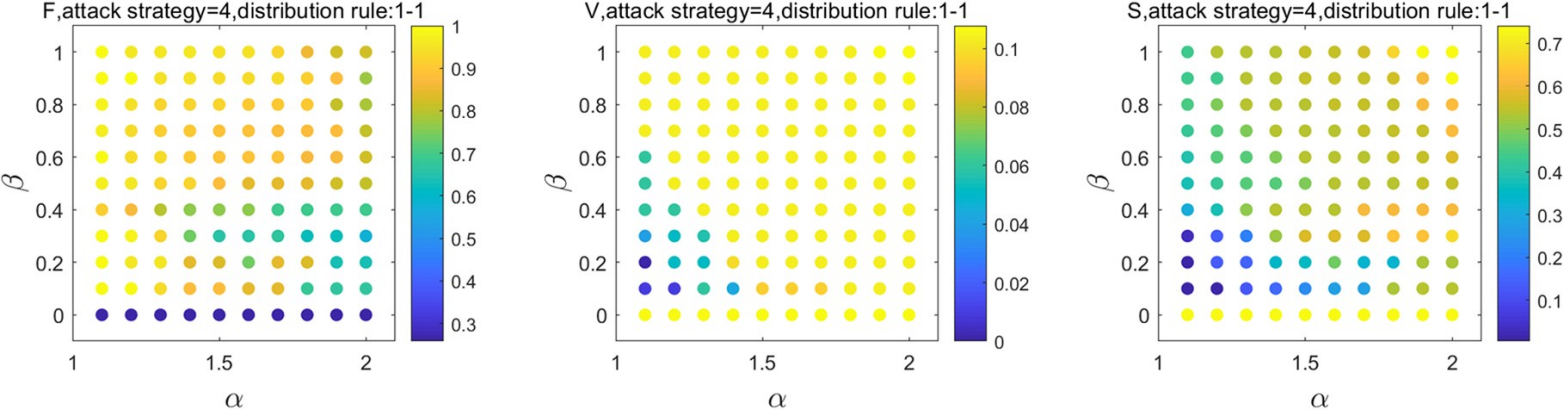
Effect of α & β on robustness of the SRN.

The reserve capacity of stations only increases within a certain range in emergencies because of the actual passenger traffic organization of the SRN. Therefore, let *β*_m_ = *β*_*r*_, *β*_m_∈[0,1], *β*_*r*_∈[0,1], *α*_*m*_ = *α*_*r*_, *α*_*m*_∈(1,2], *α*_*r*_∈(1,2], and *μ*_*m*_ = *μ*_*r*_ = 0.5. Here, *β*_*m*_, *β*_*r*_, *α*_*m*_, *α*_*r*_, *μ*_*m*_ and *μ*_*r*_ are the reserve coefficient, overload coefficient, and transfer coefficient of the MN and RRN, respectively. The effect of *α* and *β* on the robustness of the SRN is shown in Fig 3. After 30 iterations, F decreased by 23% when *β*_*m*_ = *β*_*r*_ = 0.4 and *α*_*m*_ = *α*_*r*_ = 1.4, which was the largest decrease. Second, when *β*_*m*_ = *β*_*r*_ = 1 and *α*_*m*_ = *α*_*r*_ = 2, the decrease in F is 18%, and F only decreased by 4%, when *β*_*m*_ = *β*_*r*_ = 0.2 and *α*_*m*_ = *α*_*r*_ = 1.2.

These results indicate that, the smaller the reserve coefficient and overload coefficient, the lower is the capacity of the stations, and the higher is the failure rate of stations in the overload state in the SRN. The reserve capacity, overload capacity, and F are negatively correlated. When *β*_*m*_ = *β*_*r*_ = 0.2 and *α*_*m*_ = *α*_*r*_ = 1.2, V decreases by 95% and S by 86%, with the largest decrease. Nevertheless, V decreased by 89% and S decreased by 27% when *β*_*m*_ = *β*_*r*_ = 1 and *α*_*m*_ = *α*_*r*_ = 2. The results indicate that the growth of *β*_*m*_, *β*_*r*_, *α*_*m*_, and *α*_*r*_ only increases by 6% for V; however, it increases by 59% for S. The improvement in the reserve capacity and overload capacity of stations is useful to increase the survival rate of stations in the SRN while contributing less to the network connectivity. The reserve capacity of the SRN is limited by the original capacity of the station and the cost of reconstruction and expansion; the overload capacity needs to consider the effect of the station layout and passenger traffic organization. Meanwhile, considering the capacity of the adjacent stations to accept additional load, the reserve capacity and overload capacity can only be increased within a reasonable range to ensure that the station nodes can satisfy the demands without a considerable effect on adjacent stations.

**Fig 4:**
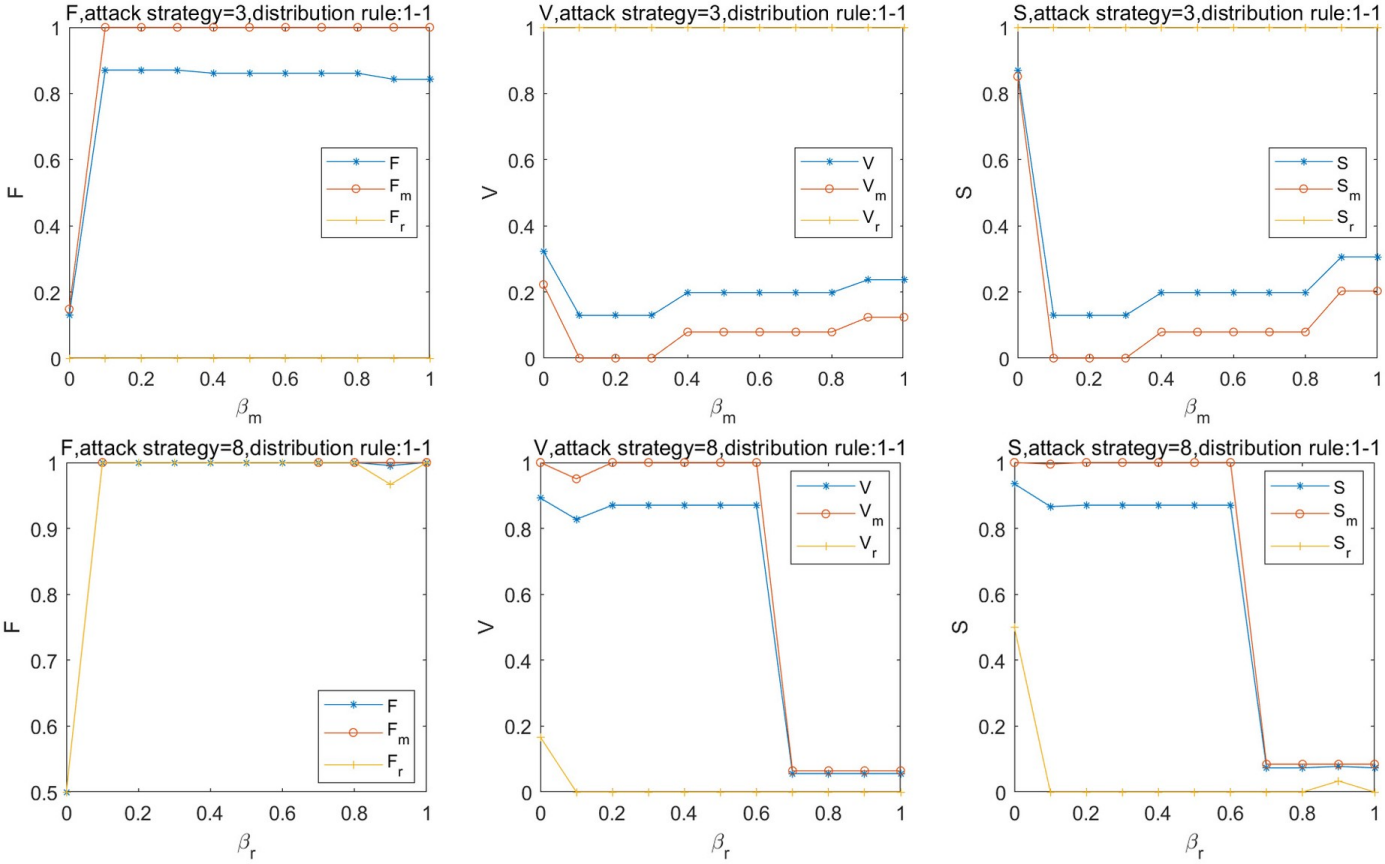
Effect of *β*_*m*_ & *β*_*r*_ on robustness of the SRN.

In practice, because of the different reserve capacity of the stations in the subnetwork, the influence of the change in the reserve coefficient on the subnetwork is not consistent. The effect of *β*_*m*_ and *β*_*r*_ on the robustness of the SRN is shown in Fig 4. The V and S decrease to 0.13, when the larger stations of the MN fail. When *β*_*m*_ = 0.4, V and S increased by 7%, when *β*_*m*_ = 0.9, V and S increased by 4% and 11%, respectively. The change in *β*_*m*_ has a great influence on the MN, which results in more failed stations caused by the overload. Although there are overloaded stations of RRN, there is no station that fails because of the overload. Therefore, to improve the reserve capacity of stations in the MN by expansion and reconstruction, it is necessary to consider the original capacity, reconstruction cost, and comprehensive benefits of the station, and to improve the carrying capacity by relocation or reduction of equipment, adjustment of entrance and exit, expansion of station hall, and addition of facilities and equipment to achieve *β*_*m*_ = 0.4. The RRN completely collapses when *β*_*r*_ = 0.1. The V and S of the SRN is 0.82 and 0.86, respectively; when *β*_*r*_ = 0.7, V and S decreased by 82% and 80%, respectively. The stations of the RRN with a high load have a great influence on the robustness of the RRN, which is easy to collapse. The change in *β*_*r*_ has no obvious effect on the robustness of the SRN. It is understandable that when the load increases to a certain range, the increase in the reserve capacity in the RRN cannot improve the robustness of the SRN significantly.

**Fig 5:**
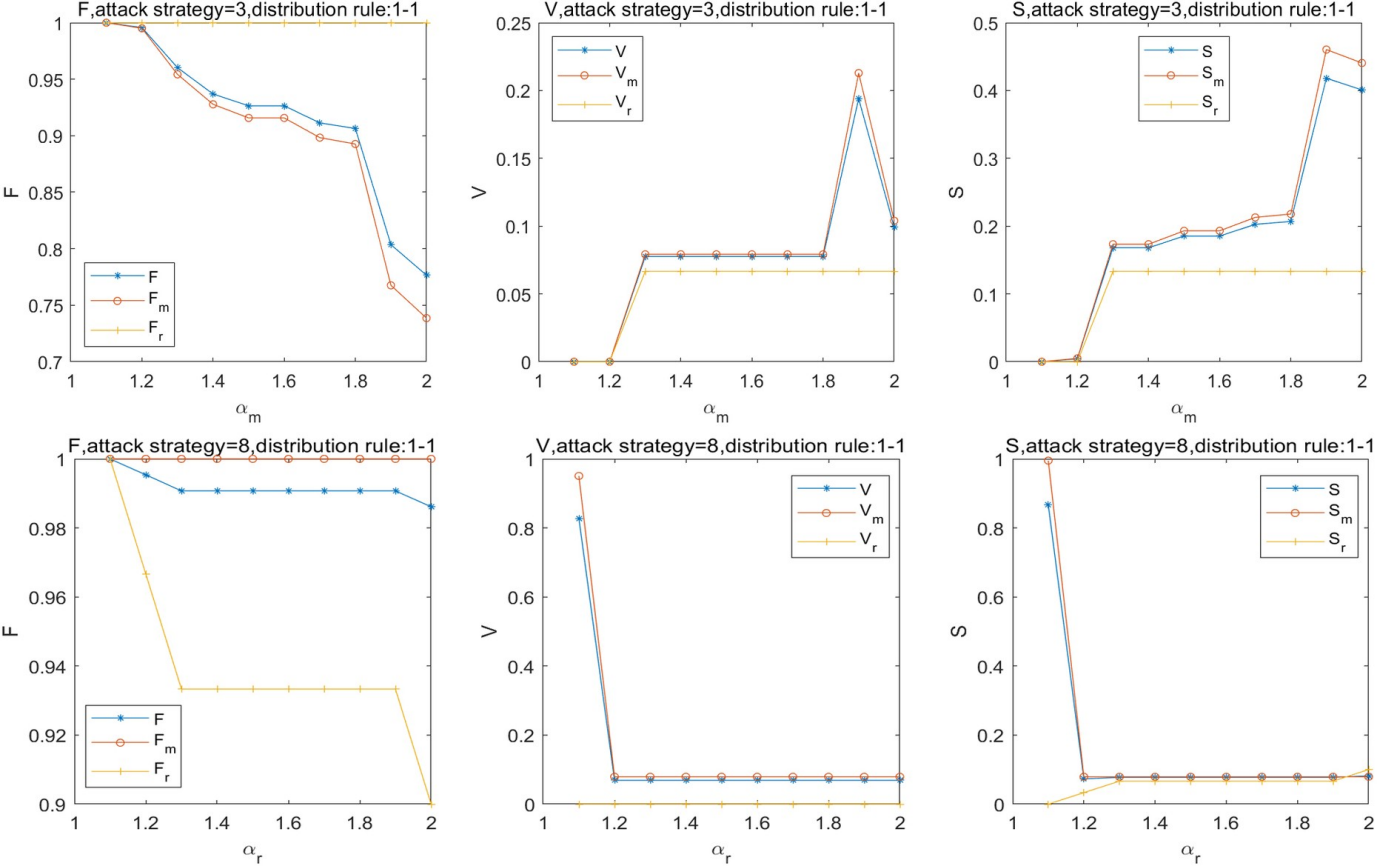
Effect of *α*_*m*_ & *α*_*r*_ on robustness of the SRN.

Furthermore, the effect of *α*_*m*_ and *α*_*r*_ on the robustness of the SRN, MN, and RRN is shown in Fig 5. When *α*_*m*_ = 1.3, V and S increases by 8% and 17%, respectively. When *α*_*m*_ = 1.9, V and S increases by 11% and 21%, respectively, with the greatest robustness of the SRN. When *α*_*r*_≥1.2, V and S decrease by 93%, respectively. The result indicates that when *α*_*m*_ is small, the load distributed to the adjacent station node from the overload station is large, and the overload station becomes full load; however, the adjacent station node is easy to overload and fail. When *α*_*m*_ is large, the overload station and the adjacent station can be easily overloaded and fail. When the load increases to a certain range, the increase in the overload capacity of the RRN does not considerably contribute to the robustness. Hence, considering the effectiveness and safety of the passenger traffic organization, the certain overload capacity of stations should be in a reasonable range and not have a considerable influence on adjacent stations. The best value of *α*_*m*_ is estimated to be 1.9.

#### Effect of *μ*

**Fig 6:**
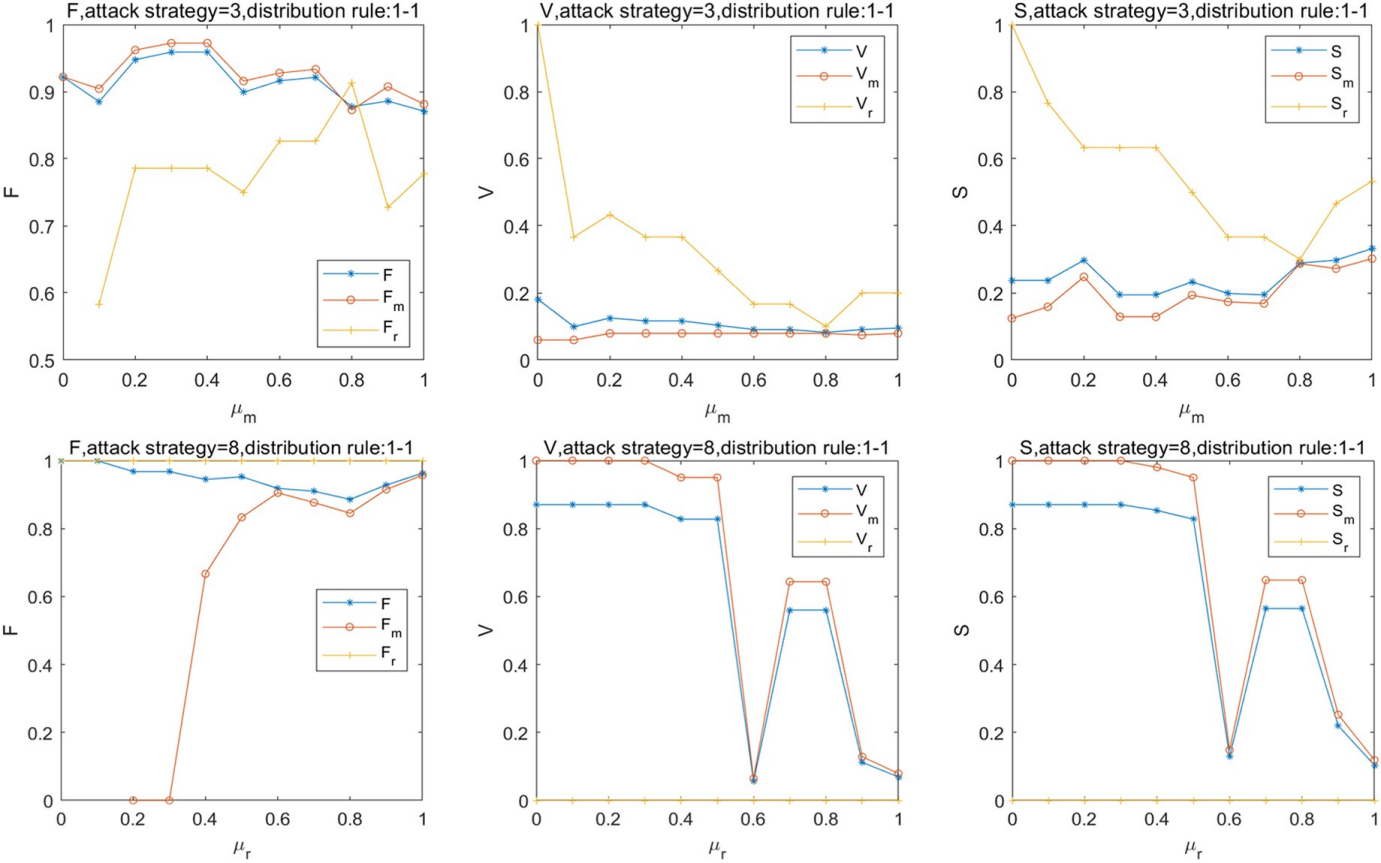
Effect of *μ*_*m*_ & *μ*_*r*_ on robustness of the SRN.

Influenced by the travel preferences of passengers and the capacity of stations, the transfer coefficient in interdependent stations between the MN and the RRN is not a fixed value. The effect of *μ*_*m*_ and *μ*_*r*_ on the robustness of the SRN, MN, and RRN is shown in Fig 6. When attack strategy 3 is adopted, V and S decreases by 89% and 75% on average, respectively. When *μ*_*m*_ increases to 0.8, *V*_*r*_ = 0.1 and *S*_*r*_ = 0.3, and the robustness of the RRN is the worst. When *μ*_*r*_ = 0.6, *V* and *V*_*m*_ decreases by 95%, S decreases by 87%, and the connectivity of the SRN and MN is the worst. The results indicate that the capacities of the MN and RRN are insufficient to accept the passenger load reallocated from each other. Meanwhile, the passenger load redistributed from the MN to RRN is insufficient to alleviate the overload failure of the critical stations of the MN. The change in *μ*_*m*_ has a stronger effect on robustness than that of *μ*_*r*_. In addition, the urban rail transit can operate normally when *μ*_*r*_≤0.5. Although the survival rate of the stations improved with *μ*_*m*_, the connectivity of the MN is too weak to transport effectively in emergencies. Therefore, to improve the robustness of the SRN, it is necessary to focus on the transfer stations inter subnetwork with a large load, i.e., East railway station and Xipu.

### Effect of load redistribution strategy on robustness of the SRN

**Fig 7:**
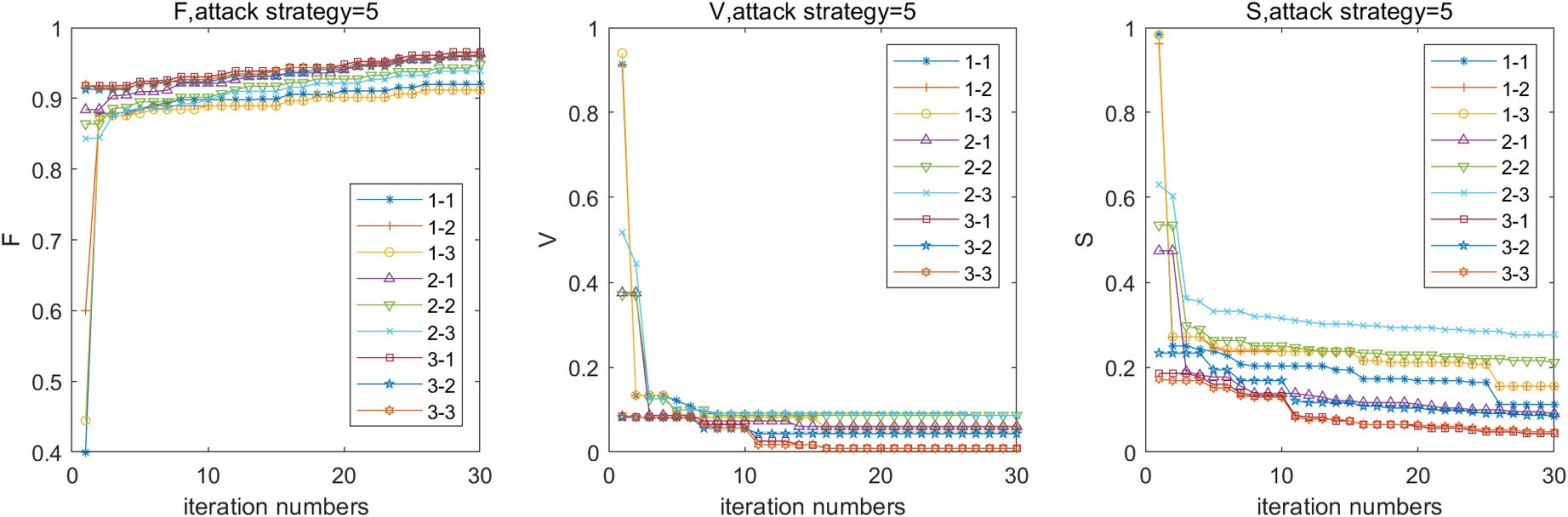
Effect of load redistribution on robustness of the SRN.

Owing to the differences in the characteristics of passenger flow, infrastructure conditions, and passenger traffic organization of each subnetwork, the load redistribution strategy of the MN and RRN are not the same. Therefore, it is more reasonable to study the effects of different load redistribution strategies on the robustness of the SRN. Let *β*_*m*_ = *β*_*r*_ = 0.5, *α*_*m*_ = *α*_*r*_ = 0.5, and *μ*_*m*_ = *μ*_*r*_ = 0.5; then, adopting attack strategy 5, the influence of different load redistribution on robustness of the SRN is shown in Fig 7 and Table 3. We use form A-B to illustrate the load redistribution of different subnetworks. A and B denotes the load redistribution strategy. For example, 1–2 represents the load redistribution of the MN is SLD and the load redistribution of the RRN is DD.

**Table 3:** Effect of load redistribution on robustness of the SRN, MN, and RRN.

MN-RRN	Iterations Number of Station	SRN				MN				RRN			
		1	10	20	30	1	10	20	30	1	10	20	30
**1–1**	In GCC	212	21	13	13	201	19	13	13	11	2	0	0
	Normal	222	26	20	8	197	24	20	8	25	2	0	0
	Full load	6	21	19	18	4	20	19	18	2	1	0	0
	Overload Fail	3	175	173	176	0	148	143	146	2	17	10	0
**1–2**	In GCC	211	19	13	13	201	19	13	13	10	0	0	0
	Normal	217	33	29	17	196	33	29	17	21	0	0	0
	Full load	6	22	20	19	5	21	20	19	1	1	0	0
	Overload Fail	8	167	163	166	0	138	133	136	7	19	10	0
**1–3**	In GCC	218	19	13	13	201	19	13	13	17	0	0	0
	Normal	223	33	29	17	197	33	29	17	26	0	0	0
	Full load	5	22	20	19	4	21	20	19	1	1	0	0
	Overload Fail	3	167	163	166	0	138	133	136	2	19	10	0
**2–1**	In GCC	87	17	14	14	76	14	14	14	11	3	0	0
	Normal	94	15	13	13	78	13	13	13	16	2	0	0
	Full load	16	17	13	8	14	14	12	8	2	3	1	0
	Overload Fail	121	190	186	181	109	165	157	151	11	15	9	0
**2–2**	In GCC	86	20	20	20	76	20	20	20	10	0	0	0
	Normal	107	39	39	39	86	39	39	39	21	0	0	0
	Full load	17	19	14	10	16	17	14	10	1	2	0	0
	Overload Fail	107	164	159	153	99	136	129	123	7	18	10	0
**2–3**	In GCC	120	21	21	20	103	21	21	20	17	0	0	0
	Normal	130	55	54	53	104	54	54	53	26	1	0	0
	Full load	16	18	14	11	15	17	14	11	1	1	0	0
	Overload Fail	85	149	144	138	82	121	114	108	2	18	10	0
**3–1**	In GCC	19	15	2	2	13	13	2	2	6	2	0	0
	Normal	26	16	2	2	17	14	2	2	9	2	0	0
	Full load	17	15	12	8	15	14	12	8	2	1	0	0
	Overload Fail	188	191	198	192	169	164	168	162	18	17	10	0
**3–2**	In GCC	19	13	10	10	13	13	10	10	6	0	0	0
	Normal	37	23	11	11	26	23	11	11	11	0	0	0
	Full load	17	16	13	9	16	15	13	9	1	1	0	0
	Overload Fail	177	183	188	182	159	154	158	152	17	19	10	0
**3–3**	In GCC	20	13	2	2	13	13	2	2	7	0	0	0
	Normal	23	14	2	2	17	14	2	2	6	0	0	0
	Full load	17	16	13	9	16	15	13	9	1	1	0	0
	Overload Fail	191	192	197	191	168	163	167	161	22	19	10	0

Furthermore, the number of stations which are in GCC, normal, full load, and overload fail is calculated, respectively. When adopting 1–1 and 1–3, 96% of the stations are in normal, and the proportion of the stations connected is 91% and 94%, respectively. Under these combinations, the robustness is the strongest. The proportion of stations in the MN that cannot transfer passenger flow to other rail transit modes is 14.3% and 12.9%, respectively. There exists a bus transit system that complements the MN for supporting urban transportation partially.

For 3–3, the proportions of the stations in the normal and connected stations are 10% and 9% respectively. The robustness of the SRN is the weakest. The proportion of stations in the MN is in cooperation with the bus transit system at 15.8%. The result demonstrates that when SLD strategy is adopted in the MN, the robustness of the SRN is the strongest. In addition, the robustness of the SRN is the worst when the BD strategy is adopted. Therefore, under abnormal conditions, SLD strategy is recommended to improve the robustness of the SRN.

### Effect and countermeasure of attack strategy on robustness of the SRN

**Fig 8:**
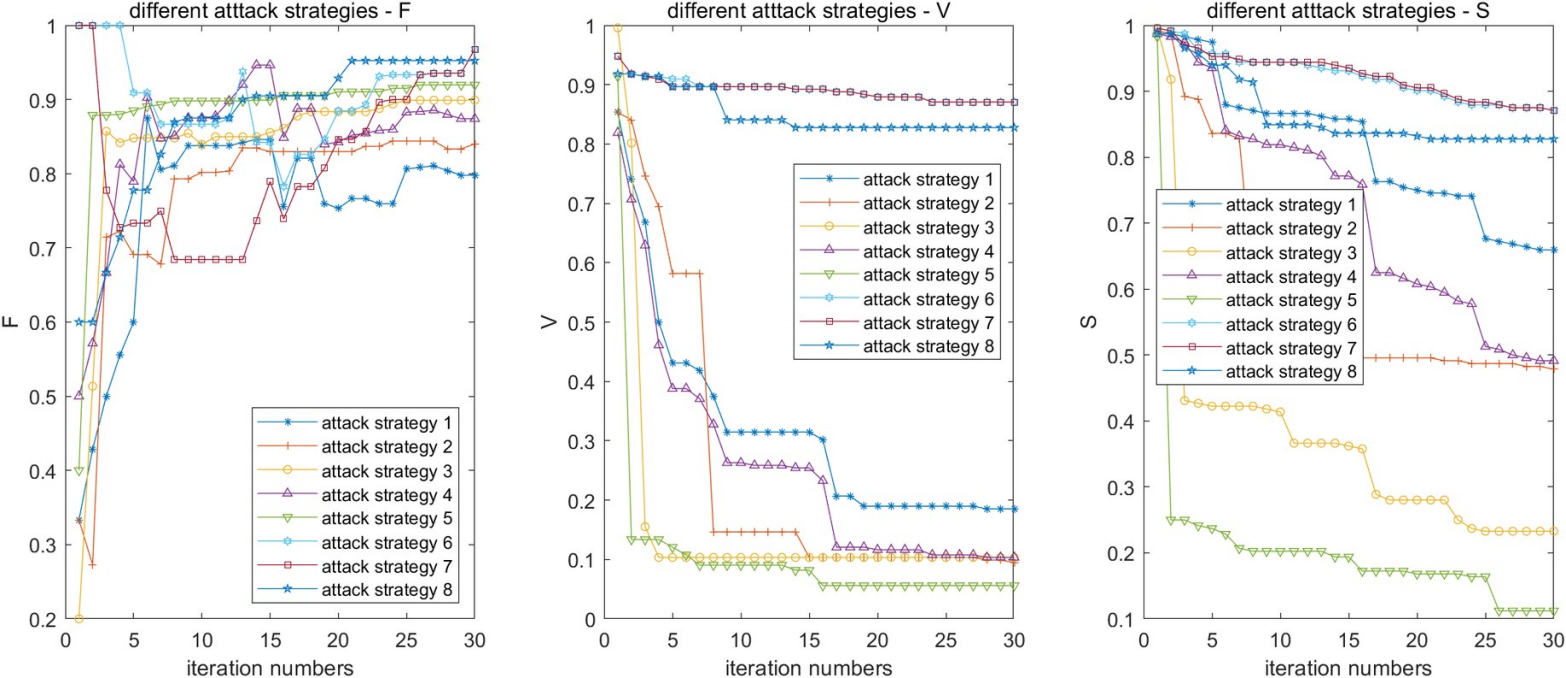
Effect of attack strategy on robustness of SRN.

Let *β*_*m*_ = *β*_*r*_ = 0.5, *α*_*m*_ = *α*_*r*_ = 1.5, and *μ*_*m*_ = *μ*_*r*_ = 0.5, the effect of the attack strategy on robustness of the SRN is shown in Fig 8.

The results show that the overload failure caused by attack strategies 5 and 3 is significant for stations with a higher load of the MN, followed by attack strategy 2. For the RRN, the overload failure caused by attack strategy 8 is obvious. We observe that the overload failure of the SRN caused by load redistribution of the MN is prominent. Meanwhile, when attack strategy 5 is adopted, three stations fail due to overload after an iteration, and only 91% of the stations are connected. Further, only 6% of the stations are in normal after 30 iterations, with the worst robustness of the SRN.

When attack strategies 6 and 7 are employed, 87% of the stations are connected. In addition, the robustness of the SRN is the strongest. This is because the number of stations and passenger load of stations in the MN is larger than that in the RRN, and the attack strategies aimed at the MN have a greater effect on the robustness of the SRN. Therefore, we need to focus on the operation organization and emergency management of the MN during peak hours. It is suggested to adopt the method of optimizing the organization, i.e., to adjust the plan of train diagram, increase the number of train sets, and compress the stop time to increase the traffic density in the peak sections and the reserve capacity in the critical sections. Second, the critical stations should ensure the capacity of the station during the peak period by adopting appropriate flow control measures, i.e., reasonable arrangement of temporary ticket office, security inspection equipment, and guiding facilities of passenger flow.

## Conclusions

In this study, a novel cascading overload failure with the tunable parameters of load redistribution inter subnetwork was developed to characterize the dynamical process of cascading overload failure in the SRN under partially coupled and overload conditions during peak hours accurately. Moreover, a simulation experiment of the SRN in a metropolitan area of Chengdu, China was conducted, and the results verified the feasibility of the cascading overload failure model. The results show that:

1. The improvement of reserve capacity and overload capacity of regional rail transit stations contributes to the survival rate of stations in the SRN; however, it does not significantly contribute to the connectivity of the SRN. When the load increases to a certain range, the reserve capacity and overload capacity of stations in the RRN do not contribute significantly to the robustness of the SRN.
2. When the reserve capacity of station in the MN is improved via expansion and reconstruction, the original capacity of the station and cost of reconstruction needs to be considered comprehensively; *β*_*m*_ = 0.4 is appropriate. The stations should have a certain overload capacity with a reasonable range of *α*_*m*_ to avoid a considerable effect on the adjacent stations. When *α*_*m*_ = 1.9, the robustness of the SRN and MN is the strongest. The capacity of the MN can only realize normal transportation under the condition of *μ*_*r*_≤0.5.
3. In the period of abnormal conditions, the SLD strategy is recommended to improve the robustness of the SRN.
4. The attack strategies aimed at the MN have a greater effect on the robustness of the SRN.

The model proposed in this paper is an improvement of the theoretical model based on actual conditions. The results of this paper have important reference significance for rational planning, structural optimization, safety design, and emergency management of SRNs under collaborative organization. However, it is worth noting that the basis for the cascading overload failure of SRNs is the critical infrastructure network and passenger flow distribution. The topology of the critical infrastructure network in different regions is inconsistent, and therefore, the parameter results obtained may be different.

In future research, based on the overload cascading failure model and robustness analysis proposed in this paper, the mitigation strategy of the SRN will be developed. The overload cascading failure model based on the mitigation strategy will be constructed to analyze the cascade dynamic mechanism with a mitigation strategy in the SRN and each subnetwork under different disturbance strategies.

## References

[1] Albert R, Jeong H, Barabási AL. Error and attack tolerance of complex networks. Nature. 2000;406(6794): 378–382. 10.1038/35019019 10935628

[2] Callaway DS, Newman ME, Strogatz SH, Watts DJ. Network robustness and fragility: Percolation on random graphs. Phys Rev Lett. 2000;85(25): 5468 10.1103/PhysRevLett.85.5468 11136023

[3] Moreno Y, Gómez JB, Pacheco AF. Instability of scale-free networks under node-breaking avalanches. Europhys Lett. 2002;58(4): 630.

[4] Dobson I, Carreras BA, Newman DE. A probabilistic loading-dependent model of cascading failure and possible implications for blackouts. IEEE Proc 36th Ann Hawaii Int Conf Syst Sci 2003: 10.

[5] Wang JW, Rong LL. A model for cascading failures in scale-free networks with a breakdown probability. Physica A. 2009;388(7):1289–98.

[6] Dou BL, Wang XG, Zhang SY. Robustness of networks against cascading failures. Physica A. 2010;389(11):2310–7.

[7] Li Z, Tang X. Robustness of complex networks to cascading failures induced by Poisson fluctuating loads. Physica A. 2019;536:120848.

[8] Buldyrev SV, Parshani R, Paul G, Stanley HE, Havlin S. Catastrophic cascade of failures in interdependent networks. Nature. 2010;464(7291):1025–8. 10.1038/nature08932 20393559

[9] Parshani R, Buldyrev SV, Havlin S. Interdependent networks: Reducing the coupling strength leads to a change from a first to second order percolation transition. Phys Rev Lett. 2010;105(4):048701 10.1103/PhysRevLett.105.048701 20867893

[10] Wang J, Jiang C, Qian J. Robustness of interdependent networks with different link patterns against cascading failures. Physica A. 2014 1 1;393:535–41.

[11] Chen Z, Du WB, Cao XB, Zhou XL. Cascading failure of interdependent networks with different coupling preference under targeted attack. Chaos, Solitons & Fractals. 2015 11 1;80:7–12.

[12] Cheng Z, Cao J. Cascade of failures in interdependent networks coupled by different type networks. Physica A. 2015;430:193–200.

[13] Gao YL, Chen SM, Nie S, Ma F, Guan JJ. Robustness analysis of interdependent networks under multiple-attacking strategies. Physica A. 2018;496:495–504.

[14] Wang J, Li Y, Zheng Q. Cascading load model in interdependent networks with coupled strength. Physica A. 2015;430:242–53.

[15] Lu Y, Chen Y, Xiong J, Chen N, Zhou B, Zhu X. Effects of group size distribution on cascading failure in partially interdependent networks. Physica A. 2019;534:120703.

[16] Su X, Ma J, Chen N, Zhu X. Cascading failures on interdependent networks with multiple dependency links and cliques. Physica A. 2019;526:120907.

[17] Wang J, Fang H, Qin X. Cascading failures on correlated interdependent networks with dependency groups. Physica A. 2019;530:121355.

[18] Wu JJ, Sun HJ, Gao ZY. Cascading failures on weighted urban traffic equilibrium networks. Physica A. 2007;386(1):407–13.

[19] Zhang L, Fu BB, Li YX. Cascading failure of urban weighted public transit network under single station happening emergency. Procedia Eng. 2016;137:259–66.

[20] Liu X. Cascading failures simulation of road networks based on bayesian network inference. J Transport Syst Eng Inf Tech. 2015;15(2):210–5.

[21] Liu XQ, Wang DR. Evolution cascading failure of road networks based on hybrid utility. Syst Eng. 2016;34(07): 104–109.

[22] Wang Z, Chen X, Li C. Identifying influence sources of cascading failure for road traffic network. China J Highway Transport. 2015;28(10):98–104.

[23] Zheng-wu WA, Jie WA, Zhong-xiang HU. Study on capacity of urban road network considering cascading failure. China Civil Eng J. 2015;48(3):121–7.

[24] Wang Z, Jie W, Huang Z. Closing strategies to control cascading failure in urban traffic networks. Syst Eng. 2016;34(2):103–8.

[25] Yin HY, Quan XF. The cascading influence law and influence scope of a failure in transportation networks. J Syst Manage. 2013;22(6):869–875.

[26] Liu ZY, Lv YB, et al, Cascading failure resistance of urban rail transit network. J Transport Syst Eng Inf Tech. 2018;18(05): 82–87.

[27] Shen L, Zhang D, Xiang Y, Wang Z, Zhang T. Simulation on survivability and cascading failure propagation of urban subway-bus compound network. J Southwest Jiaotong Univ. 2018;53(1):156–63.

[28] Li CB, Zhang S, Yang ZC, et al, Invulnerability simulation in urban agglomeration passenger traffic network under targeted attacks. J Transport Syst Eng Inf Technol. 2019;19(2): 14–21.

[29] Newman M. Networks. Oxford university press; 2018 7 4 10.1002/net.21828

[30] Stauffer D, Aharony A. Introduction to percolation theory. CRC press; 2018 12 10.

[31] Ouyang M. Review on modeling and simulation of interdependent critical infrastructure systems. Reliability Eng Syst Safety. 2014;121:43–60.

[32] Li D, Fu B, Wang Y, Lu G, Berezin Y, Stanley HE, Havlin S. Percolation transition in dynamical traffic network with evolving critical bottlenecks. Proc Natl Acad Sci USA, 2015 112(3): 669–672. 10.1073/pnas.1419185112 25552558 PMC4311803

[33] Kurant M, Thiran P, Hagmann P. Error and attack tolerance of layered complex networks. Phys Rev E. 2007;76(2):026103. 10.1103/PhysRevE.76.026103 17930100

[34] Xu Z, Zhang Q, Chen D, He Y. Characterizing the connectivity of railway networks. IEEE Trans Intel Transport Syst. 2019;21(4):1491–502.

